# Age specification matters? A multi-cohort evaluation of age specification and follow-up duration in the performance of midlife dementia risk scores

**DOI:** 10.1177/25424823261478253

**Published:** 2026-08-14

**Authors:** Md Hamidul Huque, Kaarin J. Anstey

**Affiliations:** 1 UNSW Ageing Futures Institute, 7800 University of New South Wales, Kensington, Australia; 2 School of Psychology, 7800 University of New South Wales, Kensington, Australia; 3 Brain Health and Dementia Centre, 6803 Neuroscience Research Australia, Sydney, Australia

**Keywords:** Alzheimer's disease, CAIDE, CogDrisk, comparative performance, dementia, risk assessment, risk prediction, risk tool, role of age, UKBDRS

## Abstract

**Background:**

The heterogeneous performance of dementia and Alzheimer's disease risk scores makes it difficult to identify tools suitable for supporting risk reduction and prevention strategies. Although multiple factors may contribute, we hypothesized that differences in age specification and study design are the primary contributors to this variability.

**Objective:** To evaluate how age specification and length of follow-up affect the performance of three midlife risk scores: CogDrisk-ML, CAIDE, and UKBDRS.

**Methods:**

Longitudinal data were drawn from the ARIC, UK Biobank, and Whitehall II cohorts. Participants free of dementia at baseline were included. Baseline variables were used to calculate each risk score. Risk prediction was evaluated using Cox proportional hazards models with three age specifications: UKBDRS (continuous), CogDrisk-ML (categorical: 40–49, 50–54, 55–59, 60–64 years), and CAIDE (categorical age: <47, 47–53, >53 years). Predictive accuracy was assessed using Harrell's C-statistics and 95% confidence intervals (CIs).

**Results:**

Across all cohorts and risk scores, excluding age from risk scores resulted in similar C-statistics. In age- and sex-only models, continuous age produced the highest Harrell's C-statistics (95% CI) [ARIC: 0.71 (0.68, 0.73); UK Biobank: 0.78 (0.77, 0.79), and Whitehall II: 0.75 (0.62, 0.88)], followed by categorical age specifications. Compared with continuous age, categorical specifications reduced C-statistics, with the largest reduction for CAIDE. Shorter follow-up yielded higher but less precise C-statistics.

**Conclusions:**

Age specification strongly influences dementia risk model performance, especially with shorter follow-up, with continuous age yielding the highest accuracy. Our findings emphasize that differences in dementia risk prediction performance are driven primarily by age specification and cohort age structure rather than intrinsic superiority of individual risk tools.

## Introduction

Dementia is an urgent global health crisis, affecting over 55 million people and projected to triple by 2050.^1,2^ Given the lack of curative or widely accessible treatments, the identification and implementation of effective prevention strategies have become a key public health priority.^3^ Many dementia risk factors emerge during midlife, when neuropathological changes leading to dementia typically begin,^4,5^ yet public awareness remains low.^6^ In response, several dementia risk assessment tools have been developed and validated to improve risk perception and support early identification of individuals with modifiable risk profiles, thereby guiding targeted prevention strategies.^7–11^

Despite their usefulness, considerable heterogeneity (I^2^ = 99.5%) has been observed in predictive performance, with reported AUCs ranging from 0.50 to 0.87.^12^ This heterogeneity reflects important differences in the study design and intended use (e.g., clinical versus community settings), the selection of risk factors (i.e., modifiable factors and/or non-modifiable factors such as age, sex, education, biomarkers or disease indicators), and the specification of age at baseline.

Among these factors, the age structure of the cohorts and the follow-up duration are critical to accurately predicting dementia risk. Studies based on younger populations with limited follow-up may miss late-onset cases, leading to age-related ascertainment bias.^13^ This issue is especially relevant for the UK Biobank sample, where younger population age and limited follow-up result in low dementia prevalence.^12–15^ The choice of age specification also plays a central role. Age specification is the way in which age is defined and incorporated into a study design or statistical analysis, including its use as a continuous variable or in categorized age groups for purposes such as eligibility, stratification, or risk estimation.^16,17^

Differences in whether age is modeled as a continuous or categorical variable, and the choice of cut-off points, can substantially influence predictive performance. For example, comparisons of dementia risk scores in UK Biobank have shown that age alone can outperform more complex models that also include age, largely due to differences in how age is represented relative to the underlying cohort age distribution.^18^ In this context, the continuous age only model outperformed the BDSI risk tools (continuous age specification after age 65 years), the CAIDE (categorical age: <47, 47–53, >53 years), and the ANU-ADRI score (categorical age: 60–65, 65–69, 70–74, …), where the underlying sample age range was 38–73 years. Similar results were also observed in numerous risk model comparisons.^13,19,20^ Applying models with misaligned age structures to external datasets may therefore lead to misleading conclusions,^13,19^ and artificially improve/worsen predictive performance.^14,21^ However, these studies have not explicitly highlighted age specification bias, which is driving results instead focused on apparent model superiority.

Misinterpretation may also occur when tools are applied outside their intended age groups or settings, such as using midlife scores in late-life populations, or comparing clinical tools with those for public use.^22^ While existing risk models differed in their included risk factors, we hypothesized that (i) continuous age specification would yield higher C-statistics than categorical age, (ii) the choice of cut-off points for categorical age would influence predictive accuracy, and (iii) wide baseline age ranges and short follow-up periods may inflate performance driven mainly by age. In this study, we compared the predictive accuracy of three midlife dementia risk scores, CogDrisk-ML,^14^ CAIDE,^23^ and UKBDRS,^13^ using data from three well-established longitudinal cohorts and comparing their relative performance when age was included and excluded, showing that the differences were primarily due to choices of age specification.

## Methods

We used data from three large prospective longitudinal follow-up studies to evaluate how age specification and follow-up duration affect the predictive performance of three midlife dementia risk scores: CogDrisk-ML, CAIDE, and UKBDRS. The analysis was conducted between January and July 2025 and reported in accordance with STROBE guidelines.^24^

### Data sources

Date from the UK Biobank, Atherosclerosis Risk in Communities (ARIC), and Whitehall II cohorts were considered in this study.

### The UK Biobank

The UK Biobank is a population-based cohort study with approximately 500,000 participants aged 40 to 69 years, recruited between 2006 and 2010 across the United Kingdom.^25^ At baseline, participants attended one of 22 assessment centers where, with consent, they completed questionnaires and interviews on sociodemographic, lifestyle, and health factors. They also underwent physical and cognitive assessments and provided biological samples for biomarkers and genetic analyses. Participants were followed longitudinally through linkages to national health records. For the current analysis, up to 15 years of follow-up data were available until 2022. Dementia diagnoses were derived from self-reported medical history, primary care records, hospital records, and death registries.^26^ We excluded 608 individuals with early-onset dementia (diagnosis before age 65 years) and included 287,944 individuals aged 50 to 64 years at baseline. This research was conducted under UK Biobank application #79620.

### The Atherosclerosis Risk in Communities (ARIC)

The ARIC study enrolled 15,792 participants aged 45 to 65 years between 1987–1989 from four US communities.^27^ The study is still ongoing, with dementia first assessed during Exam 5 (2011–13). For this analysis, we included 5860 participants aged <65 years at baseline who underwent dementia assessment at Exam 5. No early-onset dementia cases were present in this dataset. Dementia was diagnosed using an algorithm and ICD-9 codes, following the Diagnostic and Statistical Manual of Mental Disorders (DSM-III-R) criteria.^28^ Participants had up to 29 years of follow-up. The dataset was obtained using BioLINCC (application #13630).

### The Whitehall II study

The Whitehall II study recruited 10,308 British Civil servants aged 35 to 55 between 1985 and 1988.^29^ Twelve waves of data are available with wave 12 conducted in 2015–2016. For this analysis, wave 5 (1997–1999) was treated as baseline to align age range with ARIC and UK Biobank. Wave 5 also includes most variables required for risk score calculation, and 6516 participants aged 45 to 65 were included in the analysis. Dementia was defined based on self-reported diagnosis. The dataset was obtained and analyzed through Dementia Platform UK (Application #704).

### Prediction tools included in analyses

The risk tools/models applied to the above cohorts are briefly described below:

*Cognitive Health and Dementia Risk Reduction midlife (CogDrisk-ML).* The CogDrisk-ML includes 16 risk and protective factors such as age, sex, low education, obesity, diabetes, depression, high cholesterol, traumatic brain injury (TBI), smoking status, loneliness, physical inactivity, fish intake, hypertension, stroke, sleep disturbance, and hearing loss.^14^ Sex-specific log relative risks were pooled across ARIC and UK Biobank development datasets to create a point-based score, which was validated internally within these cohorts, and externally in the Whitehall II cohort. Detailed information on development and validation is provided elsewhere.^14^ In CogDrisk-ML, age was categorized as: 40–49, 50–54, 55–59, and 60–64 years.

*The UK Biobank dementia risk score (UKBDRS).* The UKBDRS was developed by analyzing data from UK Biobank participants aged 50 to 73 at baseline,^13^ considering 11 risk and protective factors, including age, sex, education, parental history of dementia, material deprivation, diabetes, stroke, depression, hypertension, high cholesterol, and household occupancy. The risk score was validated internally within the UK Biobank cohort and externally in the Whitehall II cohort. In this score, age (in years) was treated as a continuous variable.

*The Cardiovascular Risk Factors, Aging, and Incidence of Dementia (CAIDE).* The CAIDE score estimates 20-year dementia risk using seven midlife risk and protective factors from the CAIDE cohort aged 39–64 years using logistic regression.^23^ These factors include age, sex, education, obesity, high systolic blood pressure, high cholesterol, and physical inactivity. In this score, age was categorized using arbitrary cut-offs: <47 years, 47–53 years, and >53 years.

Supplemental Table 1 presents the risk factors and outcome definitions along with their availability across datasets.

Although many dementia risk scores are available in the literature, CogDrisk-ML, UKBDRS, and CAIDE were selected for this study because they were developed using midlife cohort data and can be implemented in both research and population settings. Although CogDrisk-ML, CAIDE, and UKBDRS were developed using a mix of independent and overlapping cohorts, they differed in how age was specified during model development: continuous in UKBDRS, and categorical in both CogDrisk-ML and CAIDE. These tools do not require biomarkers, making them suitable for use in low-resource environments. Other midlife risk scores, such as UKB-RAP,^30,31^ include clinical markers of dementia, limiting their utility for primary prevention.

### Statistical analysis

In this study, we evaluated the performance of CogDrisk-ML, UKBDRS, and CAIDE in the ARIC, UK Biobank, and Whitehall II cohorts. Risk scores were calculated using (i) all available risk factors, (ii) all factors excluding age, and (iii) only age and sex, with published risk factor coefficients as reported in the original studies. Risk factors were obtained either from the baseline visit or the nearest follow-up if baseline data were unavailable, assuming individual characteristics remained relatively stable over time. This led to some missing data (see Supplemental Table 2). Risk scores were computed by summing the points assigned to each available factor defined in the original development of CogDrisk-ML, CAIDE, and UKBDRS (see Supplemental Table 3). Missing data were imputed using multivariate normal multiple imputation with ten imputed datasets.^32^ Predictive performance was assessed using Harrell's C-statistics^33^ with 95% confidence intervals (CIs).

In addition, we also assessed hypotheses related to age specification and follow-up duration by estimating age- and sex-specific coefficients using Cox proportional hazards models fitted separately within each cohort. To assess age specification, we developed and validated age- and sex-only models using existing age specification used in the CogDrisk-ML (4 categories: 40–49, 50–54, 55–59, and 60–64 years), CAIDE (age groups <47 years, 47–53 years and >53 years), and UKBDRS (continuous age). Each cohort was randomly split with 60% for model development and 40% for validation. Age- and sex-specific Cox regression models were fitted in the development datasets and validated, and predictive performance was assessed. Differences in C-statistics between two age specifications were tested using linear combinations of model parameters.

To examine the effect of follow-up duration, we repeated the above analyses in ARIC using dementia outcomes at Wave 5 (24-years follow-up) and Wave 7 (30 years follow-up), representing shorter and longer follow-up, respectively. Again, only age- and sex-specific models were considered, and predictive performance was evaluated accordingly. All analyses were performed using Stata version 16.1 (StataCorp LLC, College Station, TX).

## Results

### Sample characteristics

Supplemental Table 2 presents the availability and distribution of covariates across the ARIC, UK Biobank, and Whitehall II cohorts. Baseline age distributions varied: most ARIC participants were aged 40–49 years, Whitehall II participants were predominantly in the 50–54 age group, and UK Biobank participants were in the 60–64 age group. Sex distribution was similar in ARIC and UK Biobank (42–44% male), but predominantly male (71%) in Whitehall II. Whitehall II also had the highest proportion of participants with tertiary education (81%), followed by UK Biobank (51%) and ARIC (44%). The prevalences of hypertension, diabetes, and high cholesterol were similar, while obesity was more common in ARIC and UK Biobank. ARIC had a higher proportion of smokers and hearing loss was lowest in Whitehall II. The mean follow-up duration was 29.1 years in ARIC, compared to 16 years in Whitehall II and 12.7 years in UK Biobank, and dementia prevalence was 19.4%, 0.8%, and 0.9%, respectively. Missing data prevalence was 6%, 38%, and 48% in the ARIC, Whitehall II and UK Biobank studies.

### Comparison of CogDrisk-ML, UKBDRS, and CAIDE across cohorts

Table 1 and Supplemental Table 4 present Harrell's C-statistics (95% CI) for the CogDrisk-ML, UKBDRS, and CAIDE risk scores across cohorts, based on complete-case analysis and multiple imputations.

**Table 1.** Estimated Harrell's C-statistics (95% CI) of CogDrisk-ML, CAIDE, and UKBDRS risk tools in dementia risk prediction (complete case analysis).

	ARIC			UK Biobank			Whitehall II		
	Male=2327; Female=3155; Total=5482			Male=69,578; Female=77,779; Total=147,357			Male=2924; Female=1106; Total=4030		
	CogDrisk-ML	UKBRDS^§^	CAIDE	CogDrisk-ML	UKBRDS^§^	CAIDE	CogDrisk-ML	UKBRDS^§^	CAIDE
Full Model with all the available risk and protective factors									
Male	0.71 (0.69, 0.74)	0.71(0.68, 0.73)	0.68 (0.66, 0.71)	0.77 (0.76, 0.79)	0.79 (0.78, 0.81)	0.58 (0.56, 0.60)	0.70 (0.60, 0.81)	0.78 (0.68, 0.89)	0.49 (0.38, 0.60)
Female	0.73 (0.71, 0.75)	0.73 (0.71, 0.75)	0.67 (0.65, 0.70)	0.77 (0.75, 0.79)	0.81 (0.79, 0.82)	0.61 (0.58, 0.64)	0.67 (0.51, 0.84)	0.69 (0.58, 0.81)	0.55 (0.39, 0.70)
Overall	0.71(0.70, 0.73)	0.72 (0.70, 0.74)	0.68 (0.66, 0.69)	0.74 (0.72, 0.75)	0.80 (0.79, 0.81)	0.60 (0.59, 0.62)	0.70 (0.62, 0.79)	0.75 (0.67, 0.83)	0.53 (0.43, 0.62)
Without age									
Male	0.62 (0.59, 0.65)	0.64 (0.61, 0.67)	0.64 (0.61, 0.67)	0.62(0.54, 0.64)	0.62 (0.59, 0.64)	0.55 (0.53, 0.57)	0.62 (0.51, 0.73)	0.55 (0.42, 0.68)	0.54 (0.43, 0.65)
Female	0.64 (0.62, 0.67)	0.63 (0.61, 0.66)	0.63 (0.60, 0.65)	0.57 (0.55, 0.61)	0.61 (0.58, 0.63)	0.58 (0.56, 0.59)	0.51 (0.33, 0.69)	0.46 (0.31, 0.61)	0.52 (0.36, 0.68)
Overall	0.61 (0.59, 0.63)	0.64 (0.62, 0.65)	0.63 (0.62, 0.65)	0.58 (0.56, 0.60)	0.63 (0.61, 0.64)	0.58 (0.56, 0.59)	0.53 (0.42, 0.65)	0.57 (0.47, 0.67)	0.50 (0.40, 0.60)
Age & sex only model									
Male	0.68 (0.65, 0.70)	0.69 (0.66, 0.71)	0.65 (0.62, 0.67)	0.73 (0.71, 0.74)	0.77 (0.76, 0.79)	0.61 (0.60, 0.61)	0.74 (0.63, 0.84)	0.79 (0.68, 0.90)	0.53 (0.52, 0.53)
Female	0.69 (0.67, 0.71)	0.70 (0.68, 0.73)	0.66 (0.64, 0.68)	0.75 (0.74, 0.76)	0.80 (0.78, 0.81)	0.62 (0.62, 0.62)	0.69 (0.56, 0.82)	0.73 (0.61, 0.84)	0.53 (0.52, 0.53)
Overall	0.68 (0.67, 0.70)	0.70 (0.68, 0.71)	0.64 (0.62, 0.66)	0.72 (0.71, 0.73)	0.79 (0.78, 0.80)	0.63 (0.62, 0.65)	0.73 (0.65, 0.81)	0.76 (0.68, 0.85)	0.58 (0.49, 0.67)

§Without parental history and Townsend deprivation.

In ARIC, C-statistics were similar for CogDrisk-ML and UKBDRS in the full model, which included all available risk factors, including age and sex. When age was excluded, C-statistics dropped to 0.61 (0.59, 0.63) for CogDrisk-ML, 0.64 (0.62, 0.65) for UKBDRS, and 0.63 (0.62, 0.65) for CAIDE.

In the UK Biobank, UKBDRS showed the highest C-statistics, followed by CogDrisk-ML, and CAIDE in the full model. When age was excluded, CogDrisk-ML and CAIDE provided similar C-statistics, but these were lower than the UKBDRS. As the UKBDRS was developed using UK Biobank data, its higher C-statistics may reflect optimism due to development in the same dataset. Further analysis with age- and sex- only models showed that continuous age (UKBDRS) performed significantly better than categorical age (p-value <0.001).

Results using the Whitehall II dataset were consistent with those using the UK Biobank. Wide confidence intervals across models may reflect the low dementia prevalence in these datasets.

Across all cohorts, CAIDE had slightly lower C-statistics than the other risk scores in the full model, but these became comparable when age was excluded. These findings suggest that CAIDE's lower predictive performance may reflect the reduced contribution of age in its algorithm. C-statistics were consistent between sexes in all models. Analyses based on multiple imputation produced similar results to complete-case analyses (Supplemental Table 4).

### The role of age in dementia risk prediction across three cohorts

Supplemental Table 5 presents the age and sex distribution in the development and validation datasets for the ARIC, UK Biobank, and Whitehall II cohorts, using three different specifications for age (continuous and two categorical). Overall, age and sex distributions were similar between development and validation subsets. Dementia cases were, on average, four years older than non-cases. The number of dementia cases increased with age in an approximately linear trend in ARIC, except in the oldest age group under the CogDrisk-ML age specification. In contrast, the UK Biobank and Whitehall II had few cases below age 53 years. Males had slightly higher dementia risk than females in ARIC and UK Biobank cohorts, while sex differences were minimal in Whitehall II.

### Effect of age specification

Table 2 presents regression coefficients and Harrell's C-statistics using three age specifications. The continuous age specification resulted in highest regression coefficient in the UK Biobank, followed by Whitehall II and ARIC dataset, respectively. The continuous age specification (UKBDRS) also resulted in the highest Harrell's C-statistics across all cohorts [ARIC: 0.71 (0.68, 0.73); UK Biobank: 0.78 (0.77, 0.79), and Whitehall II: 0.75 (0.62, 0.88)]. The categorical CogDrisk-ML specification showed lower C-statistics for UK Biobank: 0.75 (0.74, 0.76) and similar C-statistics for ARIC: 0.70 (0.67, 0.72), and for Whitehall II: 0.68 (0.56, 0.80), while the categorical CAIDE specification performed worst. The difference in C-statistics between continuous and categorical (CogDrisk-ML) age specifications was 0.01 (0.00, 0.02), 0.03 (0.02, 0.04), and 0.07 (0.02, 0.12) in the ARIC, UK Biobank and Whitehall II datasets, respectively.

**Table 2.** Regression coefficients and Harrell's C-statistics (95% CI) for different specifications of age across the ARIC, UK Biobank and Whitehall study.

		ARIC dataset		UK Biobank		Whitehall II	
		Model data (n=3512)	Validation (n=2338)	Model data (n=172,766)	Validation (n=115,178)	Model (n=3906)	Validation (n=2607)
		**β (95% CI)**	**Harrell's C (95% CI)**	**β (95% CI)**	**Harrell's C (95% CI)**	**β (95% CI)**	**Harrell's C (95% CI)**
Model 1	Continuous Age	0.14 (0.13, 0.16)	0.71 (0.68, 0.73)	0.31 (0.29, 0.33)	0.78 (0.77, 0.79)	0.20 (0.12, 0.28)	0.75 (0.62, 0.88)
	sex	0.09 (−0.06, 0.24)		0.31 (0.21, 0.41)		−0.14 (−0.92, 0.64)	
Model 2	Age		0.70 (0.67, 0.72)	Age	0.75 (0.74, 0.76)		0.68 (0.56, 0.80)
	40–49	−0.67 (−0.90, −0.44)		NA		−1.53 (−3.64, 0.59)	
	50–54	Ref=0		Ref=0		Ref=0	
	55–59	0.63 (0.43, 0.82)		2.87 (2.29, 3.44)		0.33 (−0.80,1.46)	
	60–64	1.39 (1.18, 1.60)		4.23 (3.66, 4.80)		1.47 (0.54, 2.40)	
	sex	0.10 (−0.05, 0.25)		0.32 (0.22, 0.42)		−0.12 (−0.90, 0.66)	
Model 3	Age		0.64 (0.62, 0.67)	Age	0.64 (0.63, 0.65)		0.62 (0.49, 0.76)
	<47	Ref=0		NA		NAN	
	47–53	0.64 (0.31, 0.96)		Ref=0		Ref=0	
	>53	1.66 (1.34, 1.97)		4.92 (3.79, 6.05)		1.87 (0.63, 3.06)	
	sex	0.08 (−0.07, 0.23)		0.33 (0.23, 0.43)		−0.07 (−0.85, 0.70)	

Model 1: continuous age, as used in UKBDRS, Model 2: Categorical age with 4 categories (age 40–49, 50–54, 55–59, and 60–64 years) as applied in the CogDrisk-ML, and Model 3: three age categories similar to CAIDE (<47 years, 47–53 years, and >53 years).

### The role of follow-up in dementia risk prediction

Figure 1 illustrates follow-up duration and median age of dementia diagnosis using a random sample of 200 participants across all three studies. By final follow-up, only 8% of Whitehall II and none of the UK Biobank participants reached age 80 years, the median age for dementia onset,^34^ compared to 55% in ARIC. As a result, many participants in UK Biobank and Whitehall II may not have been followed long enough to develop dementia. This age-ascertainment bias likely contributes to the lower observed prevalence and exaggerates the apparent effect of age on dementia risk. These results underscore the importance of age and follow-up duration in evaluating and comparing dementia risk prediction model performance.

**Figure 1.**
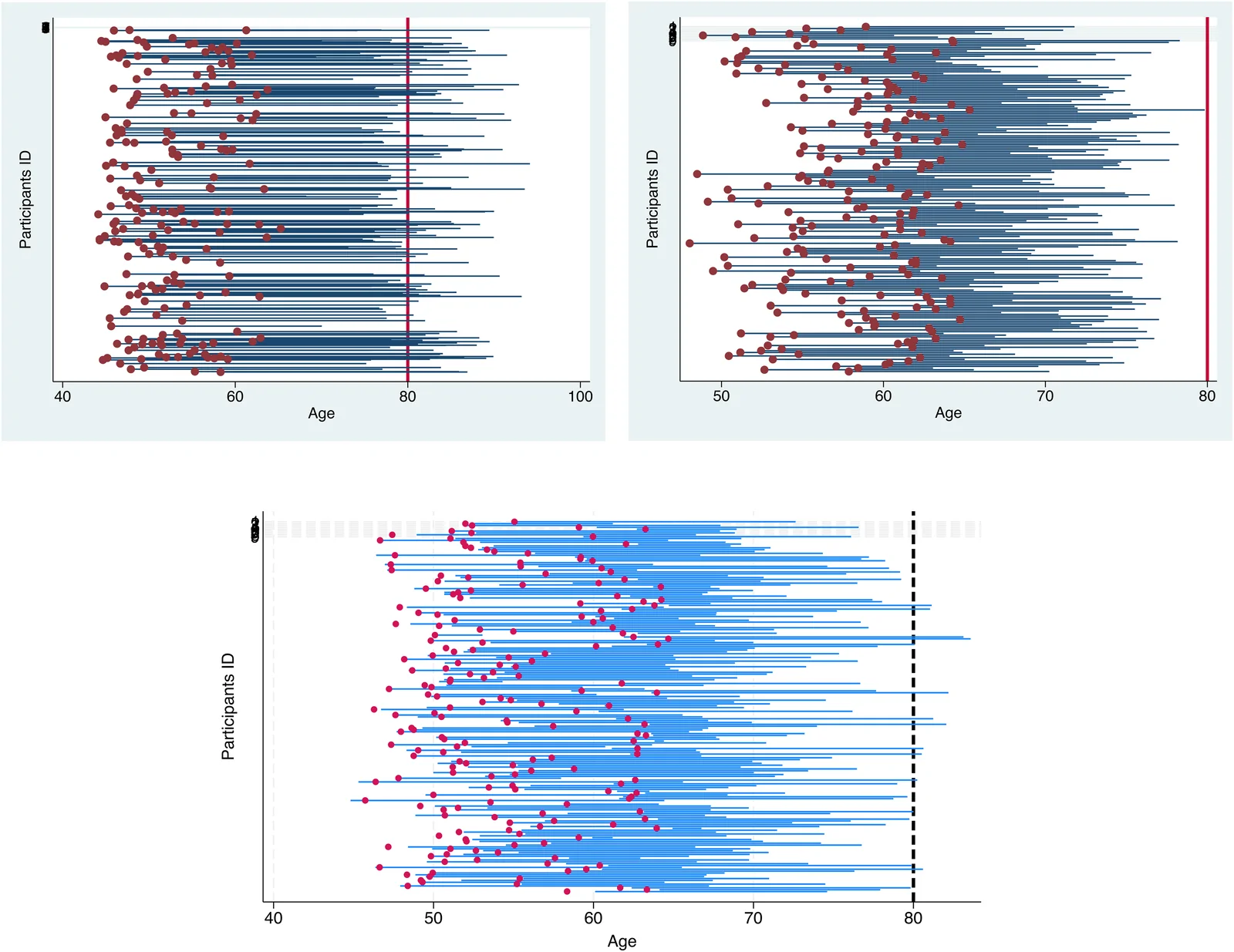
Approximate length of follow-up of 200 participants from ARIC, UK Biobank, Whitehall Cohort with respect to the median age (80 years) of dementia diagnosis. The baseline age was jittered randomly for concealment of participants in these cohorts. (Each dot at the beginning represents participant's age at recruitment and horizontal line represents the length of follow-up).

We further explored the consequence of age-ascertainment bias in the ARIC cohort, where dementia outcome was assessed at wave 5 (24-years follow-up), and wave 7 (30-years follow-up), representing shorter and longer follow-up periods, respectively. Dementia prevalence increased from 5.2% to 18.7% between these time points. The distribution of dementia outcomes by age specification and follow-up duration is provided in Supplemental Table 6.

Table 3 shows the Cox regression coefficients and C-statistics for age- and sex-only models. Coefficients were consistent across follow-up periods for both continuous age (UKBDRS) and categorical age (CogDrisk-ML), while CAIDE's categorical age produced slightly higher coefficients with shorter follow-up. C-statistics were higher for continuous and categorical age specifications (CogDrisk-ML) in the shorter follow-up, but stable for CAIDE age specification. Confidence intervals were wider in shorter follow-up, reflecting greater uncertainty due to fewer dementia events.

**Table 3.** Estimated regression coefficients and Harrell's C-statistics from Cox regression model in the ARIC development and validation dataset by follow-up duration.

		Short follow-up		Long follow-up	
		Model data (n=3512)	Validation (n=2338)	Model data (n=3512)	Validation (n=2338)
		**β (95% CI)**	**Harrell's C (95% CI)**	**β (95% CI)**	**Harrell's C (95% CI)**
Model 1	Continuous Age	0.14 (0.11, 0.17)	Male: 0.75 (0.67, 0.83)	0.14 (0.13, 0.16)	Male: 0.69 (0.65, 0.73)
			Female: 0.71 (0.65, 0.77)		Female: 0.70 (0.67, 0.74)
			Total: 0.73 (0.68, 0.78)		Total: 0.70 (0.68, 0.73)
	sex	0.10 (−0.19, 0.39)		0.09 (−0.06, 0.24)	
Model 2	Age		Male: 0.74 (0.67, 0.81)		Male: 0.68 (0.64, 0.72)
	40–49	−0.69 (−1.17, −0.21)	Female: 0.69 (0.62, 0.75)	−0.67 (−0.90, −0.44)	Female: 0.69 (0.66, 0.72)
	50–54	Ref=0		Ref=0	Total: 0.69 (0.67, 0.72)
	55–59	0.65 (0.27, 1.04)		0.63 (0.43, 0.82)	
	60–64	1.27 (0.87, 1.66)	Total: 0.71 (0.66, 0.76)	1.39 (1.18, 1.60)	
	sex	0.09(−0.20, 0.39)		0.10 (−0.05, 0.25)	
Model 3	Age		Male: 0.66 (0.58, 0.74)		Male: 0.61 (0.58, 0.65)
	<47	Ref=0		Ref=0	Female: 0.64 (0.60, 0.67)
	47–53	0.82 (0.08, 1.56)		0.64 (0.31, 0.96)	Total: 0.64 (0.61, 0.66)
			Female: 0.60 (0.51, 0.68)		
	>53	1.83 (1.12, 2.55)		1.66 (1.34, 1.97)	
	sex	0.09 (−0.20, 0.38)	Total: 0.63 (0.56, 0.70)	0.08 (−0.07, 0.23)	

Model 1: continuous age, as used in UKBDRS, Model 2: Categorical age with 4 categories (age 40–49, 50–54, 55–59, and 60–64 years) as applied in the CogDrisk-ML, and Model 3: three age categories similar to CAIDE (<47 years, 47–53 years and >53 years).

## Discussion

This study is the first to evaluate the impact of age modeling and follow-up duration on dementia risk score performance. We observed that continuous age modeling resulted in higher C-statistics than those using categorical age, particularly with shorter follow-up. Consistent with prior studies, we also observed the development sample bias, where models typically performed best on their development sample, reinforcing the need for external validation.^12^

Across all cohorts, CAIDE consistently showed the lowest predictive performance. The gap between the continuous and categorical CAIDE age specification was the largest in the age and sex only models. These findings align with those from the full models using each tool's original specification, indicating that predictive accuracy differences are primarily due to age specification rather than risk factors.

In the UK Biobank and Whitehall II, few dementia cases were diagnosed under age 53, limiting age differentiation in the CAIDE model. This highlights a limitation of applying models to cohorts outside their intended age range.^18^ Similar limitations affect late-life risk scores (e.g., ANU-ADRI,^35^ LIBRA^36^) when applied to midlife populations.^13,18^ These models were developed based on systematic reviews or expert-driven risk weights largely from older populations,^37,38^ and often lack age-sex specific points for younger individuals, result in lower C-statistics than midlife-specific models that use midlife-specific points for age and sex.^13,19^ Such comparisons may therefore be inappropriate and potentially misleading.

Across all cohorts, age and sex alone accounted for most of the variance in dementia risk. When age was excluded, performance across all models became similar. The strong performance of UKBDRS in UK Biobank and Whitehall II was largely driven by their continuous age specification. A similar pattern has been observed in studies predicting dementia using plasma biomarkers (such as NEFL, GFAP, or GDF15), which yielded modest C-statistics of around 0.70 when age was not included.^31^ However, incorporating age, sex, and education significantly improved the C-statistics in the UK Biobank data, underscoring the dominant influence of age in prediction, particularly in younger cohorts with limited follow-up.^30,39^ Because of age-related ascertainment bias in this cohort, the age only model may result in dementia risk predictions similar to those of more complex models with a large number of predictors, potentially inflating the apparent predictive power of simple age-based models.^18^

We also found that the shorter follow-up period in the ARIC cohort produced higher C-statistics but with wider confidence intervals due to fewer dementia cases than the longer follow-up period (Table 3). This finding also explains the higher AUCs observed in the UK Biobank, followed by Whitehall II and the ARIC cohort, when age was modeled as a continuous variable (Table 2). Although we analyzed ARIC data with two follow-up periods, even the shorter follow-up (24-years) was considerably longer than follow-ups used in many model development studies,^12^ warranting caution in interpreting findings from shorter follow-up duration. While datasets were harmonized as closely as possible (see Supplemental Table 1), dementia outcome and covariate ascertainment differed across cohorts. The aim of this study was therefore not to compare absolute performance of different risk tools across cohorts, but to compare the performance of the full models with age-only models and to evaluate how age specification influences predictive accuracy.

These findings have important clinical and practical implications. First, they highlight that differences in age specification can substantially influence model performance. For instance, we observed that in the UK Biobank sample, different age specifications resulted in significantly different C-statistics, with the estimated difference between continuous (UKBDRS) and categorical age (CogDrisk-ML) specifications was 0.03 (95% CI 0.02, 0.04), which largely contributed to the overall performance. Therefore, caution is needed when interpreting studies that aim to demonstrate the ‘superiority’ of a tool against other tools solely based on C-statistics and when evaluated only on the development dataset. Second, although continuous age improves predictive accuracy, it reflects a non-modifiable factor and may contribute limited value for prevention-oriented decision-making. Therefore, comparison of dementia risk scores should account for how age is modeled and weighted. While inclusion of continuous age in models produces the highest C-statistics, this approach assumes a linear risk increase across life, which may be impractical for long-term prediction.^40^ Categorical age, in contrast, may better capture non-linear patterns.^41^ In contrast, risk tools that incorporate modifiable factors and are aligned with the intended age range of the target population may be more useful for guiding early intervention strategies. And finally, our results emphasize the importance of selecting risk models that are appropriate for the population and purpose of use, particularly in public health settings where the goal is to support risk reduction rather than prediction alone.

Several limitations were identified. First, this study focuses on the methodological performance of dementia risk prediction models rather than the underlying biological mechanisms of disease and therefore does not aim to infer causal pathways between individual risk factors and dementia, but instead to inform the appropriate interpretation and application of these tools in research and prevention settings. Prevention-focused tools like CogDrisk-ML aim to identify high-risk individuals and support behavioral change. While strong discrimination is important, artificially inflating C-statistics by adjusting age specifications or thresholds offers limited clinical value.^21^ Emphasis should instead be placed on practical utility and prevention relevance. CogDrisk^42^ and CogDrisk-ML share similar, evidence-based risk factors and can be integrated into a unified tool suitable for use across midlife and late life. This life-course approach enables continuous monitoring and prevention throughout adulthood.

Second, this study did not consider machine learning based data driven models such as the UKB dementia risk prediction model (UKB-DRP) and the Florey dementia risk score (FRDS).^21,30^ With the increasing availability of large datasets, many recent models use machine learning to identify predictors that incorporate biomarkers or disease indicators such as neurological disorders, memory complaints.^21,30,39^ While clinical biomarkers may improve predictive performance and aid in detecting existing cases, they have limited value for guiding prevention-focused strategies and are therefore not directly comparable with prevention-focused dementia risk models such as CAIDE, ANU-ADRI, or CogDrisk, which aim to identify individuals at increased risk before clinical symptoms appear.

Growing evidence suggests that addressing modifiable risk factors can delay or prevent dementia onset.^43,44^ Therefore, prevention-oriented risk scores such as CogDrisk and CogDrisk-ML are valuable for identifying at-risk individuals and guiding behavioral interventions aimed at lowering long-term dementia risk. Overall, our findings emphasize that differences in dementia risk prediction performance are driven primarily by age specification and cohort age structure rather than intrinsic superiority of individual risk tools.

## Supplemental Material

sj-docx-1-alr-10.1177_25424823261478253 - Supplemental material for Age specification matters? A multi-cohort evaluation of age specification and follow-up duration in the performance of midlife dementia risk scores Supplemental material, sj-docx-1-alr-10.1177_25424823261478253 for Age specification matters? A multi-cohort evaluation of age specification and follow-up duration in the performance of midlife dementia risk scores by Md Hamidul Huque and Kaarin J. Anstey in Journal of Alzheimer's Disease Reports

## Data Availability

We have used secondary data analysis. Data may be available from data custodians.
